# A Fully Digital Auricular Splint Workflow for Post-Keloid Excision

**DOI:** 10.1055/s-0043-1771271

**Published:** 2023-08-31

**Authors:** Rahmat Maria, Yee Onn Kok, Khim Hean Teoh

**Affiliations:** 1 Department of Restorative Dentistry, National Dental Centre of Singapore, Singapore, Republic of Singapore; 2 Department of Plastic, Reconstructive and Aesthetic Surgery, Singapore General Hospital, Singapore, Republic of Singapore; 3 Department of Restorative Dentistry, National Dental Centre of Singapore, Singapore, Republic of Singapore

**Keywords:** printed auricular splint, digital workflow, ear keloid

## Abstract

Ear keloids are challenging lesions to treat due to high recurrence rates postexcision. Conservative compression techniques as adjunct treatment have been reported to be effective. An innovative technique of using computer-aided design/computed-aided manufacturing to print a customized auricular splint improves efficiency and comfort level for patients compared with conventional methods. The ear is scanned using an intraoral scanning 2 weeks postsurgery. A two-piece auricular splint is designed on the digital model, incorporating perforated projections for three nylon screws for retention of the splint. The splint is printed with clear acrylic material, postprocessed, and finished. The patient is taught to assemble the components of the splint and instructed to wear for at least 8 hours daily. The surgery site reviewed for any ulceration, pain, or recurrence of keloid for 6 months. During the 6-month review, the excision scar remained flat and pink. The patient also reports unrestricted daily activities. The digital workflow increases comfort for the patient and reduces the number of hours required to produce a customized auricular splint compared with conventional methods. A fully digital workflow for a printed auricular splint should be considered for adjunctive treatment to excision of ear keloids.

## Introduction

Computer-aided design/computer-aided manufacturing is increasingly utilized in dentistry and a digital workflow can be extended to replace conventional methods of fabricating extraoral appliances.

Keloids are challenging lesions to treat as they have a high recurrence after excision, regardless of technique. 1 2 After surgical excision, alternative adjunct procedures may include corticosteroid injections, cryotherapy, and radiation which are relatively invasive treatment options, especially for young patients.

Compression auricular splints are efficacious in preventing keloid recurrence. It has been reported that 80 to 83% of patients had no recurrence after an 18-month follow-up. 3 Various methods of fabricating ear replicas and splints have been described in the literature, many being variations of the conventional oyster-splint design and workflow by Mercer and Studd. 4

**Fig. 1:**
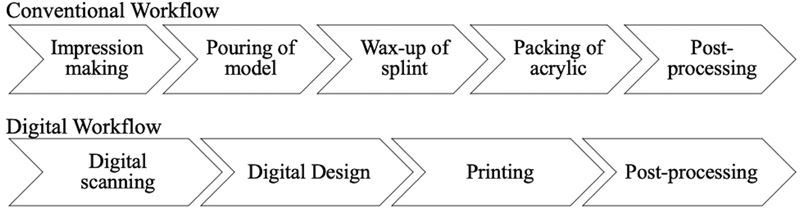
Conventional and digital workflow. A summary of the fully digital workflow described, which entails fewer steps compared with the conventional workflow. The design and wax-up of splint requires detailed planning as there are two pieces to the splint and needs to be packed in two parts. Careful removal of the splint postpacking is required as the pieces are thin and are of nonuniform shapes.

A fully digital workflow involves scanning of the ear, digitally designing, and printing of the splint. This case report describes the workflow to fabricate an auricular splint to patient post-keloid excision ( Fig. 1 ).

## Idea

**Fig. 2:**
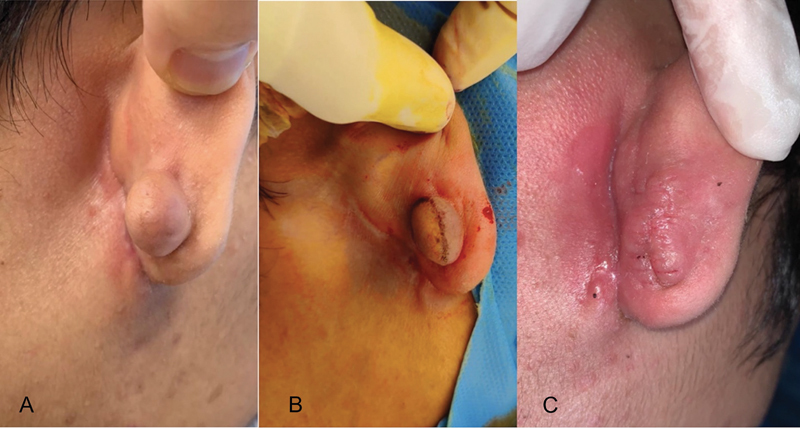
Photos of the patient's ear. Photos of the patient's right ear ( **A** ) during consultation, ( **B** ) during surgery, and ( **C** ) 2 weeks postexcision of keloid. The photos show the size of the keloid and the outcome postsurgery.

A 21-year-old male presented with a keloid of unknown cause on his right ear. The keloid was excised and treated with intralesional steroid injections, silicone gel application, and an auricular splint ( Fig. 2 ).

**Fig. 3:**
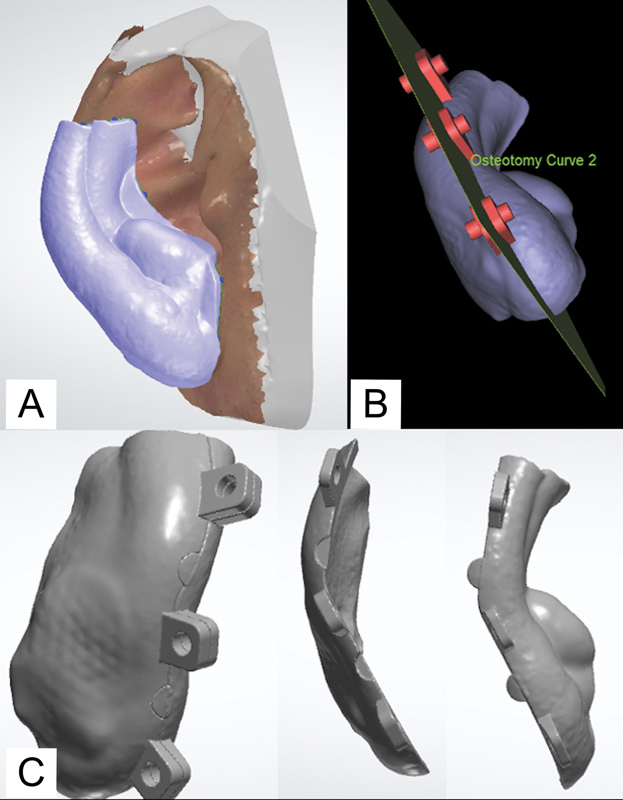
Digital design of splint. The three main steps for designing the splint: ( **A** ) a 2-mm-thick shell fabricated with planned extensions; ( **B** ) addition of three “box and cylinder” designs with an osteotomy cut to fabricate the two pieces; and ( **C** ) the final product of the two-piece auricular splint.

The patient was referred 2 weeks after excision of the keloid to the dental center. The steps for fabricating the splint were as follows:

His right ear was scanned using a portable 3Shape Trios 3 Move+ intraoral scanner (3Shape, Copenhagen, Denmark). The scanning starts with the antitragus and moves superiorly via a horizontal motion along the helix, capturing from the antihelix to the posterior of the helix. From the superior of the external ear, the scanning continues medially to the inferior crus to the concha and then the antitragus to the anterior of lobule and finally, the posterior of the lobule. The intraoral scanner provides immediate feedback on captured images and missing areas can be recaptured easily. This technique can be utilized for majority of cases except when the scanner head is too large to capture the posterior of the external ear without moving the ear. This produced a digital model for subsequent steps.
The three-dimensional (3D) model was transferred to the 3Shape Appliance Designer 2021 (3Shape) software where extensions were indicated on the digital model and a 2-mm-thick “shell” fabricated ( Fig. 3A ). This “shell” is the main body of the customized auricular splint which would later on be split into two and semicircular interlocking designs incorporated for intuitive fitting of pieces.
At least three nylon screws are required to clamp the final two-piece auricular splint together. This is done by importing the “shell” body into Proplan CMF 3.0 software (Materialise NV, Leuven, Belgium) where “box and cylinder” designs were positioned at the predicted mating surfaces of the splint. This design provides perforated projections for the nylon screws to fit and be tightened with nuts ( Fig. 3B ).
The enhancement of the design, such as smoothening and subtracting of the surfaces to complete the design, was done in Geomagic Freeform Touch software 2016.2.62 (Geomagic, 3D Systems, NC).
The “shell” was split by using an osteotomy cut ( Fig. 3B ) for the final product ( Fig. 3C ).
Finally, the two pieces were separately printed with clear acrylic (NextDent Ortho Clear, Nextdent B.V., Vertex Global Holding, Soesterberg, The Netherlands). The printed parts were cleaned with ethanol for no more than 5 minutes and set to rest for at least 10 minutes after. Postcuring was done in the NextDent LC-3DPrint Box (Nextdent B.V., Vertex Global Holding). The support structures were removed and polished.

The design of the splint was a modification of the oyster splint described by Mercer and Studd. The splint extended at least ⅔ of the external helix. It included the scar with a margin of 3 mm to ensure adequate coverage and relieved the external auditory meatus. No relief spacer was incorporated and each piece required their own path of insertion. Two nylon screws, each close to the inferior and superior borders, and one additional nylon screw in the middle, were planned in the design. This ensured adequate clamping and that the pieces would not open up when tightened. The splint was printed with a clear acrylic as it enables visualization of the wound site for complications and amount of pressure applied. The splint had cleansable surfaces for hygiene purposes.

**Fig. 4:**
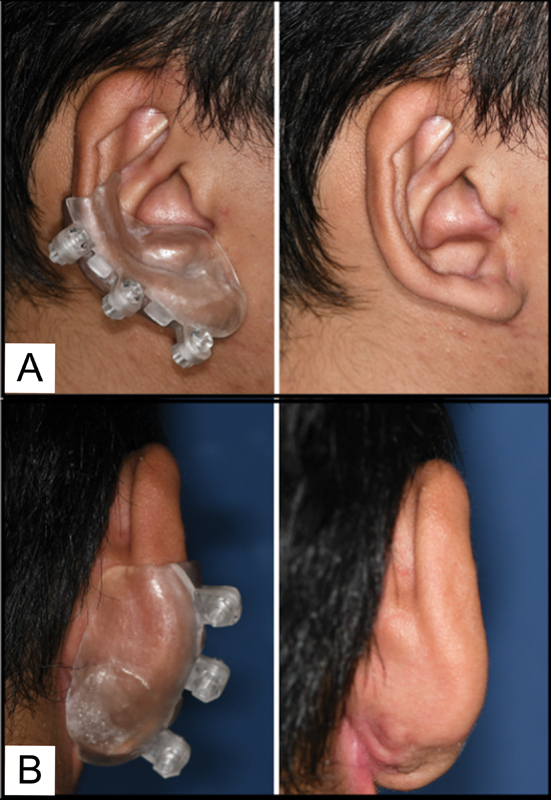
Photos of the splint in situ. The patient at 6-month review, ( **A** ) with the auricular splint and ( **B** ) without the auricular splint.

During the issue of the splint, the fit was checked, with rough or sharp areas adjusted accordingly. Retention and pressure of the splint was tested to ensure comfort. The patient was able to fit the splint and attach screws independently and wore it for at least 8 hours for the first 2 weeks and subsequently, throughout the day. Weekly reviews were done for the first month to detect any pressure-induced erythema early. Subsequently, 3- and 6-month reviews of the appliance and surgical site were done. The patient has since worn the splint for 6 months ( Fig. 4 ) and the scar remains flat and pink. In a questionnaire provided, he reported ease with handling the splint, unimpaired appearance, and unrestricted daily activities.

Instructions provided to the patient are as follow:

1. Start daily application of the silicone sheet 2 weeks after surgery if well healed.
2. Clean the surgical wound gently with a damp cloth after removing the silicone sheet.
3. During the issue of ear splint, adjustments would be made to ensure comfort and to practice fitting the splint on.
4. The splint should be worn over the silicone sheet for at least 6 hours, or as long as there is no discomfort. It is possible to sleep with the splint as long as it is not too uncomfortable.
5. Cleaning of the splint should be done every 1 to 2 days by using a damp cotton bud, with soap if necessary. Both the outer and inner surfaces should be cleaned.

## Discussion

The treatment for keloids is challenging and a combination of treatment modalities produces the best results, especially for larger keloids. For larger keloids on the ear, in addition to surgical removal, adjunct treatment such as corticosteroid injections, cryotherapy, radiation, and compression therapy have been described. Cryotherapy is effective for smaller keloids but often causes hyperpigmentation. 5 Keloid irradiation is successful, however, there have been isolated reports of skin tumor development. 6 7 8 As such, adjunct treatment modalities have potential complications, and a conservative approach of compression splint therapy is increasingly recommended to patients. Hassel et al found that 80% of patients felt the auricular splint treatment absolutely painless, with the remaining 20% finding it minimally painful. 3

The key challenge in producing a customized splint is obtaining a replica of an ear to fabricate the splint on, owing to the unique shape an ear. In the conventional workflow, a physical model is made with either an alginate or an additional polymerized silicone impression material which can be messy, but more importantly, uncomfortable for the patient. Hair gets tugged and incorporated into the impression despite a recommended layer of Vaseline coat over pulled-back hair around the ear. In addition, tugging of the ear and the recent surgical site is unavoidable when removing the impression from the ear due to the multiple undercuts of the ear anatomy. A fully digital workflow eliminates the physical process of impression-making. The comfort level improves as the scanner head moves around the patient's ear without contact.

Another large advantage with utilizing a digital impression is the immediate feedback that digital scanning provides. During the scanning process, the software captures and stitches images in real time and the clinician can check the 3D digital model on the monitor immediately. The scanning process can be paused, 3D digital model checked, and additional details of deficient areas can be scanned and captured immediately. In contrast, a conventional model with inadequate pertinent features captured would require a remake of the whole impression.

The conventional method of fabricating a splint requires a high level of mastery and is time-consuming. The impression has to be carefully prepared, poured, and then removed from the model to preserve the integrity and prevent breaking of the model. There is detailed planning involved in packing the various layers of the acrylic splint in the flask to ensure that deflasking the cured acrylic would be straightforward and the thin acrylic parts would not break. The total amount of hours required from model pouring stage to postprocessing in the conventional workflow would easily exceed 10 hours while the digital workflow potentially at least halve it. In addition, the painstakingly made model would be destroyed during the process of extracting the splint and cannot be reused. In the case where a patient misplaces or requests an addition splint, the splint can be easily reprinted if a digital workflow is utilized, compared with starting from scratch with the conventional workflow. In the center, the cost of printing an ear splint is comparable to conventionally fabricating one.

In the literature, various designs of compression therapy and appliances have been described. 3 4 9 10 11 12 13 The above design adapted from the oyster splint has various advantages incorporated. The components of the main splint are sleek and follow closely to the contours of the ear, with minimal projections, which are only for the nylon screws. This provides a more aesthetic and compact appliance which would be increased acceptability with patients, in turn increased compliance. The nylon screws provide adjustable pressure onto the tissues and reduce the chance of ulcerations and pressure sores compared with the use of magnets and clips. A minimum of three screws are required to produce an even pressure onto the tissue surface within the splint and prevents the splint from opening up as the splint is tightened. The design also incorporates a relief of the external auditory meatus to ensure that patient's hearing and daily activities are minimally impeded.

Compared with the digital workflow described by Nejat et al, the scanning protocol that has been described here does not require any indicators or markings on the ear, improving the patient's experience. Our design starts with a shell that covers the entire relevant areas that is subsequently split into two. This allows very accurate piecing of parts and allows incorporation of interlocking designs to make fitting the pieces more intuitive.

This treatment modality is not without limitations. The workflow requires a clinician familiar and trained with using intraoral scanners and 3D digital designing, with a laboratory which is familiar with 3D digital designing, printing, and postprocessing of the splint. While keeping the design compact and simple, the assembly of the splint requires the patient to have a certain level of dexterity, especially since direct vision during assembly is not possible. The patient should have a family member or a friend present during the issue visit to understand the sequence of assembling the splint in the event the patient requires assistance outside the clinic setting. A detailed discussion on the rationale of treatment is necessary for the patient to understand that compliance is key for the treatment to be effective. Studies have described utility hours of up to 23 hours a day; however, there could be discomfort when patients are sleeping. A daily 6 to 12 hours recommended wear was advised. This reduces the need of wearing and removing the splint too many times a day, potentially improving compliance.

A fully digital workflow for a printed auricular splint should be considered for adjunctive treatment to excision of ear keloids.
